# Supplementary material for: The impact of comorbid spinal pain in depression on work participation and clinical remission following brief or short psychotherapy. Secondary analysis of a randomized controlled trial with two-year follow-up
Source: PLoS One. 2022 Aug 22;17(8):e0273216. doi: 10.1371/journal.pone.0273216 (PMC9394798; doi:10.1371/journal.pone.0273216)
Supplement: Attachment — Submitted filename: PLOS ONE Review.docx [file pone.0273216.s001.docx]

PLOS ONE Review: The impact of comorbid spinal pain in depression on work-participation and clinical recovery following brief or short psychotherapy. Secondary analysis of a randomized controlled trial with 2-year follow-up

Thank you for the opportunity to review this interesting and timely paper. The paper addressed the much-needed area of how pain is related to outcomes of psychotherapeutic interventions. The paper was predominantly well written and added much interesting commentary on returning to work and the authors should be commended for their careful statistical analyses and the length of the follow up to their original pragmatic RCT. Such longitudinal follow-ups are insightful and often uncommon.

Despite many real strengths in the paper, my recommendation is, unfortunately, a rejection. Principally this is because I hold significant concerns about the validity of the identification of the pain group as a Chronic Spinal Pain group. These are concerns that the authors also note in their limitations section (See Lines 552-557), but do not seem to have mitigated or to considered in greater depth. The authors argue that their analyses are between non-chronic spinal pain and depression, representing a subsample of the original RCT study. However, the rationale for the CSP is, to my mind, quite problematic. They conceptualised the CSP group as people who scored as having some/seriously affected neck, lower or upper back pain and combined this with a question about having musculoskeletal complaints for at least 3 months in a row. Unfortunately, the musculoskeletal complaints selection did not specifically screen for back pain/spinal pain and included a wide range of body areas (inclusive of arm, pelvis, foot, etc.) and therefore it is likely that the sample may represent a huge range of people struggling with pain that may or may not be spinal. For example, people with conditions as varied as chronic pelvic pain, broader chronic pain syndromes or osteoarthritic pain in areas other than the spine could all meet the inclusion criteria. The choice to categorise this as a single chronic spinal pain group seems slightly spurious and feels as though it may not be a robust decision. I would encourage the authors to revisit their inclusion and exclusion criteria and think through the viability of the group inclusion/exclusion criteria, such that they are stronger and more justified. Given this, I am concerned that the validity of the conclusions and analyses and the way in which they are argued in relation to spinal pain are not as strong as is needed for publication. The group does not clearly settle within back pain, nor is it spinal cord injury and the clarity of the inclusion/exclusion is not adequate.

Further comments:

The abstract seems not to have populated fully on the form, losing the conclusion, but this may be because it is a long abstract and would benefit from being reduced, with fewer presentations of statistics and a broader overview presented. The conclusion is, however, present at the start of the manuscript. The abstract seems unusually lengthy.

Mental clinical recovery is a slightly unusual choice of terminology. The authors could present this as mental health, mental wellbeing or psychological wellbeing as those phrases would be more familiar to a psychological readership. Clinical recovery is a useful term but is associated with a much broader set of medical implications than is intended in this manuscript.

Introduction:

- Line 52: a reference would be beneficial to evidence your statement on LBP prognosis.
- Grammar and phrasing in lines 53-55 need revisited.
- Line 58: effect sizes would be usefully added here such that we can see the extent of the ‘smaller’ size that you mention.
- Line 60: a brief overview sentence of your original findings would be useful here for readers unfamiliar with your original paper
- Line 62: it would be helpful to explain here how the focus changed.
- Line 64: the word choice of ‘induces’ seems inappropriate here – somatic comorbidity cannot induce psychotherapy sessions.
- Lines 67-71 detail the hypothesis about why short term psychotherapy may be advantaged over brief psychotherapy, but this seems to directly contrast with the findings from your earlier study that you discuss in line 62. It seems odd to then hypothesise the opposite to what your previous work has suggested. This discrepancy should be resolved or discussed and acknowledged.
- Line 74: the substantial subgroup that is referenced here is unclear – I presume you mean the CSP group, but it would be helpful to be more specific.

Methods:

The methods in general did not feel well organised and struggled to read independently as a standalone paper separate from the previous publication. As such, the following recommendations may help the authors clarify this section:

- Line 87: Please briefly explain how your previous study was a ‘pragmatic’ RCT.
- Use of hyphens seems unusual throughout the manuscript and hyphens should be checked throughout.
- Lines 94-99: This section seems to re-read like an abstract, giving results. This is odd here and this section should just factually describe (in brief) the study design and setting.
- Please relist the materials used in the original study and provide a full materials section
- Line 105: it would be more patient-centred to refer to disorders as mental health disorders rather than mental disorders (which implies problematic cognitive function or stigmatising viewpoints).
- In the participants section, please present inclusion criteria before exclusion criteria and clarify the exclusion criteria. What counted as ‘acute or more severe pathology’? It would be useful here to report the processes and judgements that led to exclusion.
- Why was the cut-off for sick leave 9 months? This feels like an arbitrary figure and no rationale for this cut-off was presented.

Intervention:

- I query the meaningfulness of the quantitative difference between a median of 5 hours in the brief intervention and 7.5 hours in the short intervention - this is only a marginal difference in number of therapeutic hours. This needs further consideration and a rationale/justification for separation as different intervention types.
- What were the decisions around concluding or ending the intervention? Why was there such variation in number of hours within the protocol – the checks around fidelity/manualisation/standardisation of the intervention delivery are not clear in this manuscript (though I accept that this may not always be possible within pure psychotherapy). Further detail and consideration is needed about description of the intervention and the associated screening/delivery/termination.

Measures:

This should be factually presented either as a materials section (as mentioned above), where questionnaires included in the study are detailed here each in turn. Or could be presented as a primary and secondary outcome measures section. It is unusual to have DVs and IVs presented here – these might be better located in the design section in a much briefer form. Indeed at the end of your manuscript you refer to your ‘secondary outcome measures’ on line 564, but we don’t know what these are from your current presentation of the manuscript.

In general, there are a huge number of measures in this study which are contained in various sections across the method and it is difficult to see in a coherent way what was tested, on which scales and the reliability/validity of those measures and subscales. Reliability and validity of the measures in your study and how they compare with expected clinical reliability/validity checks should be presented.

Please present a Statistics and Data Analysis Section in which you fully detail your data management methods, what you did to mitigate against missing data, and your analytic strategy. The ‘potential covariates’ section is unusual and dense, containing much information that should be in the materials section (e.g. scoring of the BAI) and information about how you ran the study (which should be in the procedure section). This should be collapsed with your statistical analyses section and some significant care taken to present data management and analysis decisions clearly and concisely in a single location within the manuscript.

Line 172: please present some statistical validation for the use of the adapted version – does the internal consistency match the original measure?

Why are particular questions selected for inclusion in analysis rather than using full questionnaires? E.g. line 192? The selection of individual questions raises concerns about the validity and robustness of the data when repeatedly treated in isolation from the measures as a whole.

A huge number of outcome measures were treated dichotomously rather than as interval variables. The decisions around where the dichotomous split was chosen to be located seems random and no justification is given or checks about the appropriateness of this. Indeed Line 213 states variables were dichotomized according to the median or other ‘pragmatic decisions’ but these are not detailed. It is not appropriate just to opt to create splits simply for pragmatism. Referenced and robust decisions should inform such choices. I would recommend that, where possible, creation of dichotomous variables should be avoided and the authors should consider alternative methods of analysis, including Bayesian statistics (to account for small subgroups) or bootstrapping.

Line 187: please use quotation marks for “my job is…

Statistical Analyses:

Lines 196-197: why are the hypotheses repeated here? This is not needed.

Line 198: what is meant by baseline variables were ‘participanted’?

Lines 201-204: please report the data check outcomes. Which measures failed and on which tests? What was done about missing data? Was missing data MNAR or MCAR? Little’s test would be useful here.

What is the rationale for the prediction models? How are these theoretically-informed? It would be useful to justify these models more clearly in the literature that you review in the introduction.

In general, crude odds ratios should be avoided if possible and authors should aim to prioritise adjusted odds ratios.

Results:

Effect sizes and confidence intervals should be presented throughout – these are not consistently presented. Please ensure they are present either in tables or throughout the manuscript. Please italicise p-values.

Lines 280-282: Grammar seems unusual in this sentence.

The language in the results section can sometimes be tentative, suggesting that the authors are underconfident about stating the results strongly. E.g. line 341 ‘seemed to predict’ or 334 ‘seemed thus not a significant dilemma’. Please check throughout.

Discussion:

Line 381: avoid the contraction of did not.

Throughout the discussion, the authors restate statistical findings and use statistical language. I would suggest that the authors revisit the discussion section in full and aim to avoid re-presentation of statistical findings and instead focus on the (clinical) implications of their findings and their relationship with psychological/medical literature and psychological theory. By reducing the amount of time spent revisiting the statistics, there would be greater space for linking the findings with previous literature, which would be more informative for the reader and would locate the manuscript more strongly within the literature base.

It would be useful to have further elaboration of the mechanisms and theoretical justification for the findings, particularly around lines 427 and 428. There is minimal inclusion of theory-driven explanation in the manuscript at present.

Lines 459-460. This is an interesting finding and it would be helpful here to have more discussion about why correlations with depression but not anxiety might have occurred.

Line 467: check grammar in ‘worth to consider’ (worth considering?)

Line 492: if you are presenting the lack of association as interesting, it would be great to see more speculation/literature-based explanation about this rather than suggesting it is out of scope.

Lines 506-512: It is possible that the moderating impact of CSP on psychotherapy could be muted by the lack of differentiation between the intervention types/hours and also by the lack of clarity of the recruitment strategies. This needs discussed.

Lines 519-522: Pain acceptance is not typically seen as a physiotherapy issue. This is more usually dealt with in Acceptance and Commitment therapy which is psychology led. Therefore I don’t feel that the physio consult would have induced acceptance and coping, so this section feels weak.

It may be also useful to think through the extent to which your results may reflect the fact that there could be an issue here in terms of stratification of patients to the ‘best’ treatment approach. For example, it is possible that the ‘CSP’ group may have shown better outcomes if they were first seen within pain management services rather than in talking therapies. This research may suggest that this stratification/screening should occur.

Line 552: I strongly agree with this comment and feel this problematises the manuscript as a whole.

Conclusion: It would be helpful in the final paragraph (586-588) to have an additional sentence about the meaning of the fact that the impact could not be confirmed in the current study. An explanation of the importance of a null funding is of equal importance as the findings that were positive.
