# Supplementary material for: The impact of comorbid spinal pain in depression on work participation and clinical remission following brief or short psychotherapy. Secondary analysis of a randomized controlled trial with two-year follow-up
Source: PLoS One. 2022 Aug 22;17(8):e0273216. doi: 10.1371/journal.pone.0273216 (PMC9394798; doi:10.1371/journal.pone.0273216)
Supplement: Attachment — Submitted filename: Response to Reviewers.docx [file pone.0273216.s003.docx]

Dear Reviewer,	April 22 2022, Stavern Norway

We appreciate the time and effort that you have dedicated to providing valuable feedback on our manuscript. We have considered all comments and incorporated changes and/or motivations to reflect most of the suggestions provided.

We see that comments were mainly related to three main concerns:

- First, our attention to comparing the two subgroups at baseline and to apply this to identify relevant covariates (confounders) as (s)he indicated earlier as well. We have tried to improve the reasoning for this even more.
- The second concern was the additional aim of the current study to evaluate whether the occurrence of comorbid spinal pain brought about effect modifications following the two different interventions. We concluded on this, but apparently, we had not made this clear enough as a defined aim of the study.
- Third, the reviewer suggested to remove, shorten or undo some removals of paragraphs in the revised manuscript. However, this mainly concerned suggestions from the other reviewers and we have chosen to keep with these in case we considered these as improvements to our first manuscript.

Throughout the manuscript we have strived to be more clear by using correct and consistent terminology. When aiming at the impact of comorbid spinal pain on long-term outcome we focus on CSP as a ‘prognostic factor’. In the evaluation whether the two interventions has differential effect on the patients with or without comorbid spinal pain we use the term ‘effect modification’. During the process of selecting other baseline characteristics as ‘covariates’ we label these as ‘potential cofounders’.

Below are comments to the suggestions of reviewer 2 and a point by point account of changes made in the revised manuscript. The *black text in italics* are the comments by the reviewer while our comments are in blue and the changes in the manuscript are marked in green.

***Abstract***

*The abstract is more readable, however, you should keep the estimates in the result section (not delete them as suggested in the revised manuscript). As previously highlighted, there is still inconsistency between the aim of the study and the conclusion.*

The estimates had been removed after earlier suggestion of another reviewer to reduce presentations of statistics and rather present a broader overview. We have kept us to this, especially because of the number of words in the abstract.

We apologize for not taking this issue seriously enough in the former revision and have improved the abstract, particularly ‘objectives and ‘conclusions’:

“Objectives: This explorative study analyses the influence of baseline comorbid long-lasting spinal pain (CSP) on improvement of long term work participation and clinical remission of mental health illness following either brief coping-focussed or short-term psychotherapy for depression. Whether type of treatment modify outcome with or without CSP is also analysed.

“Conclusions: CSP at baseline reduced work participation and worsened remission of mental health symptoms two-year following psychotherapy. Older age and more severe baseline anxiety are associated to reduced effectiveness. Type of psychotherapy received did not contribute to differences in intervention effects.”

***Introduction***

*Line 112-117: Suggest that you describe the aim of the study as research questions rather than hypothesis-testing.*

We have rewritten the aim of the study as research questions and removed our hypotheses and objectives:

“The main research question of the current study is to determine whether baseline CSP is prognostic for long-term sick leave and reduced clinical remission of mental health problems following psychotherapy for depression. Second, it is questioned whether CSP may moderate the effect of brief versus short psychotherapy. To answer the first question, we will evaluate whether CSP is an effect modifier for improvements on sick leave and clinical remission of mental health problems two-year following psychotherapy for depression and to estimate the magnitude of that effect, if any. To enable adjustment for confounding, we need to examine the differential associations between CSP and other baseline characteristics and their association with intervention outcome as well. Secondly, we will evaluate whether the effects of brief-coping focused and short psychotherapy with a more extended focus are equally beneficial in participants with baseline CSP compared to participants without CSP, or not.”

***Method***

*Line 130-131: Not necessary to write the aim in this section (already described). Here you also write “primary” aim. If you have a primary and secondary aim, this should be described clearly.*

The primary and secondary aim mentioned in this section referred to the aims of the primary RCT. We acknowledge that this can be confusing and removed this sentence.

*Line 280: Not clear what you mean by “potential baseline covariates”. These are descriptive variables, and not variables that you want to include in statistical analysis according to your aim*

*Line 289-292: It is not clear how the described statistical tests comparing the subgroups are needed in relation to your aim. And what it add to the conclusion of the study. Suggest to remove this.*

*Line- 298-231: I would not recommend to base the inclusion of possible confounders in the model on statistical tests, rather base this on a clinical and theoretical foundation.*

As our focus is on the impact of CSP on intervention outcome we need to adjust for possible confounding baseline variables. These confounders are the variables that are both related to baseline CSP and affect intervention outcome and we need to include those in our model as covariates. The descriptive variables were pre-selected for their expected potential of being a confounder based on literature review and our clinical experience.
In the process of selecting relevant covariates as described in step 1 and 2 of the development of the prediction model, we had labelled them as ‘potential baseline covariates’. However, in this revised manuscript we have tried to make it more clear why it is essential to perform some analysis with these to evaluate the statistical associations of these descriptive variables with both the dependent variable CPS (step 1) and with the independent intervention effect variables (step 2) as well. We therefore changed the wording from “potential baseline covariates” to “potential confounding baseline variables” to make it more precise what the actual role of the covariates in the model are.

“Potential confounding baseline variables were selected based on a review of the literature concerning work participation and clinical outcome in common mental health or spinal complaints (table 1). In this context, confounding variables are both related to CSP and affect intervention outcome. The included baseline variables were subjected to descriptive statistical analysis stratified for the presence of CSP. Data of both intervention arms were pooled. Their relationship with both CSP and intervention outcome were evaluated as described below in the selection of covariates for the regression models.”

To examine the prognostic and effect modifying impact of CSP, two separate models were tested, one for each treatment response variable: WP2yr and CR2yr. Each prediction model was developed in five consecutive steps. Step 1 involved selection of potential confounders as covariates, based on the results of the association of the initial selected baseline variables with CSP. To be comprehensive we chose a lenient level of differential significance for including possible covariates (p.15). In step 2, analyses (Chi-squared test with Yates’ continuity correction for all 2x2 analyses and Mann–Whitney U test for numerical variables) were applied to assess the associations of these variables with baseline WP and treatment response for both defined binary dependent outcome variables: WP2yr and CR2yr.

***Results***

*Line 362-375: Can this section be shortened?*

We acknowledge that you commented on this same section in your previous review. Unfortunately, we did not shorten this section as a compromise with one of the other reviewers who requested even more information. We think it is informative.

*Line 377 -> : This section is not specified as an aim, nether as suggested hypothesis, and I don’t see the relevance in performing al these statistical tests. Suggest to remove or rephrase the aim of the study.*

*Table 4: Still not related to the aim of your study. You only describe in the aim that you are interested in the predictive ability of CSP, and not anything about identifying possible prognostic factors in general. This should be aligned. Or the information/results could be put in supplementary files.*

I think we have specified the importance of including this section and the statistics above. In the description of the study aim we included “To enable adjustment for confounding, we need to examine the differential associations between CSP and other baseline characteristics and their association with intervention outcome as well.”
And we start the concerning section ‘Comparative characteristics according to the presence of comorbid spinal pain’ with “To assess the balance between the groups at baseline with respect to the main prognostic or confounding factors, relevant baseline measures stratified for the presence of spinal pain (subgroup non-CSP vs subgroup CSP) are presented in Table 1.”

***Discussion***

*Section from 550 ->: This section can be shortened, as it is not the main aim of you study to identify the differences between participants with and without CSP.*

As mentioned previously, we think this section is strongly relevant to the main aim of the study to identify the specific effect of CSP on intervention effect. In this, it is necessary to assess what the relevance is of the differential characteristics of this subpopulation. We have added:

“Confounding is by definition a nonissue in RCTs, but in the comparison of intervention effects within subgroups of a RCT it is essential to assess possible confounding factors that are related to both the occurrence of spinal pain and the intervention outcome measures.”

*Line 656-665: Text needs to be written more clear, and better relate it to the context of your study.*

We have rewritten this paragraph and hope it will be more clear and related to the study. The new test read:

The present findings of higher baseline anxiety in CSP, rendering the impact of CSP on symptom remission as insignificant, is complementary to previous research reporting negative prognostic value of baseline anxiety on pain outcome [76, 83, 85]. The causative mechanism behind the association is not clear and may include several pathways. Reviewing this, Gureje [60] claims that the pattern of association supports a causal pathway that proceeds from mental disorder to chronic pain rather than the reverse. In a longitudinal study among individual with chronic pain it was found, indeed, that neither pain nor pain-related disability predicted depression/anxiety [86]. Conversely, symptoms of both depression and anxiety, prospectively predicted levels of pain and pain related disability. However, findings from a large longitudinal twin study [87] found that pain predisposed for depression or anxiety symptoms, indicating a relationship explained by shared pathophysiological pathways. Possibly, the coexistence of anxiety and pain may point to a reciprocal relationship in which the direction might be dependent on context. Negative health cognitive bias, shared neurobiological features and somatization processes may mediate this relationship [86]. Based on the results of our present study, we will limit ourselves to informing clinicians that pain and anxiety may covaries and that both would have to be addressed in therapy.

*Line 680-682: You argue that fear avoidance are equally distributed in both subgroups, but you report the baseline value as median. Is it not possible that the individual variation within the subgroup still may influence the association?*

We agree that fear avoidance behaviour in principle may be an obvious differential variable comparing the subgroups with and without CSP. As higher scores indicate that the individual has elevated fear-avoidance beliefs towards work, we may expect higher FABQ-work scores in the CSP group because of the additional burden of having CSP. In general, a total FABQ-work score higher than mean 4.85 is considered as a high, positive FABQ-test result.

We interpret your comment to suggest that the median may be misleading in the sense that the within group distribution may still vary so much that a group difference is actually present, but not visible by comparing the median performance of the two groups. The median score presented here is the median of the individual mean scores of all FABQ-w items. However, we have also reported (see table 1) the interquartile ranges of this measures that showed quite similar variance within the subgroups (for FABQ-w 1.5-4.1 vs. 1.1-3.9).  In the analysis comparing the median, it is important to stress that there were neither any close to significant tendency for group differences with regard to fear avoidance (p=.35). Thus, we think it is defendable to state in the Discussion part that there were no differences.

*Limitations: You still don’t thoroughly describe how the limitation in the study may influence the specific results of the analyses. A discussion of the sensitivity analyses can for instance be relevant here.*

We have included in the limitation section:

“Although subgrouping was based on a pre-randomized baseline characteristic, a point of concern is that the decision to perform subgroup analyses was post hoc. The RCT was therefore possibly not adequately powered to do so. Consequently, the probability of generating ‘false-negative’ results in the analyses in this current study are substantial.”

…. “Although sensitivity analysis for subgroup are especially relevant for post-randomization groups, we did however, perform sensitivity analyses to see how redefining the threshold for subgroup defining changed the observed subgroup and intervention outcome differences. For all four variants, contrasts and level of significance between subgroup outcome was greatest in the subgrouping that is reported here.”

***Conclusion***

*You highlight the research question regarding the short vs. long intervention. This should also be more clear in the aim of the study.*

We hope it is clearer now from the defined research questions that the study aims at both assessing the influence of baseline comorbid long-lasting spinal pain (CSP) on intervention improvements and in addition at examining the possible treatment effect modification.

Thank you for giving us the opportunity to once more revising our manuscript.

Best regards

Marjon Wormgoor
